# RACIAL DISPARITIES IN RURAL-URBAN MORTALITY GAP IN THE UNITED STATES: A 24-YEAR LONGITUDINAL STUDY

**DOI:** 10.1093/geroni/igz038.2685

**Published:** 2019-11-08

**Authors:** Nasim Ferdows, Soroosh Baghban Ferdows, Amit Kumar

**Affiliations:** 1 University of Southern California, Los Angeles, California, United States; 2 Istanbul Technic U, Istanbul, Turkey, Turkey; 3 Northern Arizona University, Flagstaff, Arizona, United States

## Abstract

Although overall life expectancy in the US has improved rapidly over the course of the 20th century and the racial gap in all-cause mortality has declined in recent decades, geographical disparities in mortality have increased in the last three decades. This research aims to study racial and geographical disparities by comparing the race and sex-specific mortality trends of the US rural and urban populations. We created a longitudinal county level analytic file of the US population 65 years and older, over the period of 1968 to 2015 obtained from CDC-WONDER and Area Health Resources Files. First, we used an OLS regression of age-adjusted mortality rate onto year indicators interaction with race and gender to depict the race and sex-specific trend in age-adjusted mortality rates. We also estimated the change in in mortality rate over time, for each race and gender, relative to values in 1968. Finally, we estimated race and sex specific trend in rural-urban mortality gap using state fixed effects regression. Our results indicate that racial gap in mortality rates has only declined in urban areas. Mortality rates of the whites in rural areas declined more rapidly than their Black counterparts, resulting in a gap that has been widening in the last three decades. The racial gap has increased considerably for males residing in rural counties not adjacent to an urban county. Thus, racial disparity in mortality has increased in rural areas, with a considerable widening between white and black male population living in the more remote rural areas.

